# Role of calcium phosphate and bioactive glass coating on in vivo bone healing of new Mg–Zn–Ca implant

**DOI:** 10.1007/s10856-021-06510-0

**Published:** 2021-05-07

**Authors:** Arnab Mahato, Munmun De, Promita Bhattacharjee, Vinod Kumar, Prasenjit Mukherjee, Gajendra Singh, Biswanath Kundu, Vamsi K. Balla, Samit Kumar Nandi

**Affiliations:** 1 Bioceramics and Coating Division, CSIR-Central Glass and Ceramic Research Institute, Kolkata, India; 2 Department of Veterinary Surgery and Radiology, West Bengal University of Animal and Fishery Sciences, Kolkata, India; 3 Materials Science Centre, Indian Institute of Technology, Kharagpur, India; 4 Veterinary Clinical Complex, West Bengal University of Animal and Fishery Sciences, Mohanpur, Nadia India; 5 Muve Hospital, Surat, 395007 Gujarat India

## Abstract

Present investigation focuses on development and detailed characterization of a new Mg alloy sample (BM) with and without coating of hydroxyapatite (BMH) and bioactive glass (BMG) by air plasma spray method. After detailed mechano-physico-chemical characterization of powders and coated samples, electrochemical corrosion and SBF immersion tests were carried out. Detailed in vitro characterizations for cell viability were undertaken using MG-63 cell line followed by in vivo tests in rabbit model for studying bone healing up to 60 days. Starting current density increases from BM to BMH to BMG indicating highest resistance towards corrosion in case of BMG samples, however BMH also showed highest *i*_corr_ value suggesting slowest rate of corrosion than BM and BMG samples. Dissolution of calcium ion in case of BMH and BMG control formation of apatite phases on surface. Ca^2+^ ions of coatings and from SBF solution underwent reduction reaction simultaneously with conversion of Mg to MgCl_2_ releasing OH^−^ in the solution, which increases pH. Viability and propagation of human osteoblast-like cells was verified using confocal microscopy observations and from expression of bone specific genes. Alkaline phosphatase assay and ARS staining indicate cell proliferation and production of neo-osseous tissue matrix. In vivo, based on histology of heart, kidney and liver, and immune response of IL-2, IL-6 and TNFα, all the materials show no adverse effects in body system. The bone creation was observed to be more for BMH. Although both BMH and BMG show rays of possibilities in early new bone formation and tough bone–implant bonding at interface as compared to bare Mg alloy, however, BMG showed better well-sprayed coating covering on substrate and resistance against corrosion prior implanting in vivo. Also, better apatite formation on this sample makes it more favourable implant.

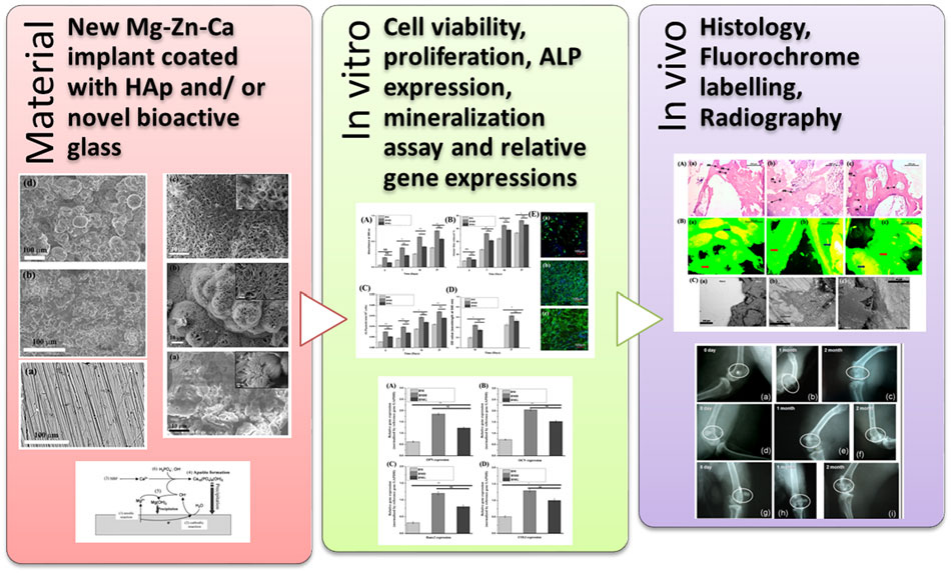

## Introduction

Magnesium alloys gathered special interest in recent years in context of structural lightweight applications in temporary implants because of their ability to be gradually dissolved, absorbed, consumed or excreted through urine [1, 2]. High load bearing capacity and fracture toughness compared to existing biodegradable polymers made them more suitable as orthopaedic implant. Biodegradability of these alloy implants implies that they need not be removed through second surgery, thus minimizing trauma and medical expenses [3]. Recent investigations have used Mg alloy based wound closures for gastrointestinal procedure [4] and polymer coated Mg alloy based scaffold for sustained drug release [5]. However, the major obstacle against widespread application is their high corrosion rate [6, 7], which might be attributed to the presence of different impurity elements acting as active cathodic site when in solution. Presence of elements such as Fe, Ni, Cu and Co above their tolerance limit may significantly increase Mg corrosion [8]. Whereas presence of elements such as Ca, Zn, Si and Al up to a certain quantity can improve the mechanical properties as well as corrosion resistance [9, 10]. Depending up on the requirements of biomedical applications, magnesium alloys has been modified through various methods such as surface modification via anodization [11], electrodeposition [12], chemical conversion coatings [13] or organic coatings [14] or alloying. In case of biodegradable implant application, alloying seems to be preferable method as ion release will maintain a continuity. Among the Mg alloy systems, Mg–Ca and Mg–Zn have shown a balance of properties, mechanical and corrosion resistance, suitable for biomedical applications [15]. Calcium helps to improve bone healing process, whereas Zn is known to increase the mechanical strength of the alloy [16]. Inspired by the result, Kirkland et al. prepared and examined a series of ternary Mg–Zn–Ca alloys by varying the ratio of Zn to Ca and came to the conclusion that the corrosion is correlated with alloy microstructure, which can be varied through changing the ratio [17]. Recent studies on Mg–Zn–Ca alloying system show high mechanical properties, simultaneous high strength and high ductility (yield stress, R_p0.2_ > 250 MPa, ultimate tensile strength R_m_ > 300 MPa and fracture strain, A_f_ > 20%) within a composition window of 5–6 wt.% Zn and 0.2–0.4 wt.% Ca [18, 19]. Studies by Mao et al. showed that with increasing Zn content, microstructure is more refined and mechanical properties are improved [20]. Depending on the results, most researchers have assumed that ternary alloys based on Mg–Zn–Ca system are safe for in vivo use. In vivo evaluation of Mg–Zn–Ca system demonstrated no sign of inflammation 4 weeks after first implantation when used in 3-month-old C57BL/6 mice [21]. Another study using Mg_60_Zn_35_Ca_5_ in pig also supported the utility of Mg–Zn–Ca system in the field of implant application [22]. Park et al. modified the last composition (Mg_62_Zn_35_Ca_3_) to use calcium as a grain refinement agent, whereas Zn to increase mechanical strength. Results confirmed better biocompatibility and improved corrosion resistance as the samples were absorbed completely after 4 weeks from implantation without any inflammatory phase [23]. Thus, adequate amount of Ca and Zn can be added to pure Mg to improve biocompatibility and mechanical property without showing toxicity [24]. According to the available data, it is yet to formulise the effect of Zn, Ca or Zn/Ca ratio in ternary alloy systems. However, to ensure long-term stability, alloying never proved to be enough due to degradation caused by electrochemical corrosion by formation of cathodic sites in microstructure. In order to elongate the stability, degradation must be slowed, in other words corrosion resistance must be increased. Thus, several procedures have been used to retain mechanical properties for longer period, among them surface coating predominates due to its simplicity yet effectiveness towards the requirement. Various physical, chemical, mechanical and biological deposition methods have been adopted for depositing bioactive glass (BG) and bioceramic on various substrates [25]. It was suggested that surface coating of Mg-based implant has advantages of gradual degradation over a period of time along with superior corrosion consistency enabling strength decay [26]. And in this regard, advantages of plasma spray technique include its ease of preparation, coating on unique surface with good adhesion, conformal and pin-hole free films, less leachability, sterile upon preparation and in case of magnesium alloy, less chance of corrosion by atmospheric moisture.

Further, surface modification of Mg alloys can be classified into metallic, ceramic and polymeric according to the chemical characteristics and atomic structure of the coating materials, reviewed by Yin et al. [27]. Choice of coating material has become a topic of interest in many research groups. Different materials such as ceramics and glass (hydroxyapatite (HAp), calcium phosphates, 45S5 bioglass, etc. [28–31]), polymer (PGA, PLA, PEO-PCL, PEA-PLLA, PLG, etc. [32]) and composite (HAp-PCL, F-HAp [33]) have been used according to the implant requirements. CaP coatings done by ion-beam-assisted deposition or electrochemical and chemical treatment reduce the rate of corrosion, but crystal structure, chemical composition, coating morphology and degradation rates differ. Sometimes less cell viability on the longer run was also reported [34]. However, according to the substrate material, component design, cost and end applications, coating thickness requirement and process temperature, plasma spray coating was chosen as one of the coating procedures. And further, keeping the variables in mind BG and HAp were chosen as coating material in the present investigation. BGs when implanted react to physiological fluids and form a strong chemical bond with bone. It forms a hydroxyl carbonate apatite layer on the surface and delay further corrosion. Use of HAp as coating material in hybrid structures and metals has gained attention due to interaction of HAp with tissue [35].

Thus, in the present study, we have developed and used a new ternary system (Mg–Zn–Ca) of Mg alloy based substrates, which was further coated using air plasma spray system with HAp and for the first time with BG S53P4 as well as coating material. Aim of this study is to understand the compositional effect of substrate on coating in long-term use and finally suitability as implant material for bone defect healing in animal models for 60 days.

## Materials and methods

### Fabrication of samples

#### Sample preparation

In the present investigation, we have used a new Mg alloy with alloying elements of ~22.5% Zn and 0.5% Ca by wt. was used as substrate material (henceforth, the samples will be referred as BM). Alloying elements were quantified by X-ray fluorescence spectroscopy (XRF) prior. Further, phases of alloys were analysed by X-ray diffraction (XRD) (PANalytical, the Netherlands) and Fourier-transformed infra-red spectroscopy (FTIR) (Spectrum 100, PerkinElmer, USA) and scanning electron microscopy-energy dispersive analysis of X-ray (SEM-EDAX) (Phenom proX, Phenom-World B.V., the Netherlands) was used for microstructural and approximate qualitative elemental determination. Samples were cut into rectangular strip/plate with size 100 × 10 × 3 mm (*L* × *b* × *t*) using abrasive cutting machine. Surface of the specimens was roughened by 99.9% high pure alumina grit (16 mesh) using a pressure blast (MEC Shot Blasting Equipments Pvt. Ltd., India). Roughness of bare surface was kept around 6–10 μm (average). Finally, acetone was used to clean all the samples ultrasonically and dried at room temperature for further use.

BG S53P4 was synthesized by conventional melt-quench method using SiO_2_ (Loba Chemie, Mumbai, India, Min. assay 99.7%), CaCO_3_ (Min. assay 98.5%), Na_2_CO_3_ (Min. assay 99.9%) and (NH_4_)_2_HPO_4_ (Min. assay 99%) (Merck, Mumbai, India) as raw materials. Calculated batch were mixed thoroughly and melted at 1360 °C followed by quenching in double distilled water. Frits were collected and extensively milled at 250 rpm using a planetary ball mill (PM100, Retsch, Germany) followed by further grinding and sieving to obtain granules ranging between 70 and 150 μm. The powder chemical composition was checked by wavelength dispersive XRF spectrometry. The final powder was also tested to ensure particle size distribution (Microtrac S3500, USA).

On the other hand, HAp was prepared by conventional wet chemical method using A.R. grade calcium hydroxide [Ca(OH)_2_, Central Drug House, India] and ortho-phosphoric acid (H_3_PO_4_, Merck, India) as raw materials. Stoichiometry of reagent materials was maintained in a way (1.67) that pure phase can be obtained. After completion of drop-wise mixing of H_3_PO_4_, solution was kept for 24 h for precipitation. Precipitate was then washed and filtered followed by drying at 80 °C for 24 h. After drying, this was ground and sieved to get homogeneously sized powders. Powder was fired at 800 °C to obtain phase pure HAp. Finally, the same was graded sieved to obtain granules ranging between 70 and 150 μm. Sintered (at 1250 °C) free flowing granules were used for subsequent plasma spray coating purpose. Further details have been reported elsewhere [36].

Strips/plates of BM were used for HAp and BG coating by using air plasma spray system (Sulzer Metco, USA) (henceforth, BG and HAp coated BM samples shall be referred as BMG and BMH, respectively). Six-axis manipulator (ABB Engineering, China) was used to obtain uniformity of the coating on substrate. Plasma cathode, a conical tip, was made of thoriated tungsten, while copper was used to make anode/nozzle of the torch with a conical shape that finished in a cylindrical duct 6 mm in internal diameter. Torch generation was carried out using argon (primary plasma gas) and hydrogen (secondary plasma gas). Spray conditions used are detailed in Table 1.

**Table 1:** Spray condition used for plasma spray coating of HAp and bioactive glass on Mg alloy

Coating parameters	HAp coating (BMH)	S53P4 coating (BMG)
Arc current	500 A	350 A
Current	458 A	357 A
Voltage	62.2 V	58.2 V
Argon	55 NLPM	55 NLPM
Hydrogen	3 NLPM	2 NLPM
Flow rate	21 g/min	28 g/min
Water conductivity	39.9 µs	39.9 µs
Gun distance	6 inch	6 inch
Average time	40 s	35 s

#### Feedstock powder and coating characterization

Phase analyses of the powders were carried out by powder XRD with Cu Kα (λ = 1.54178 Å) radiation [40 kV/30 mA with 2θ between 20° and 60°, step size of 0.05°] and further verified by FTIR [KBr pellet method; mid-IR range of 4000–400 cm^−1^, resolution 4 cm^−1^] spectroscopy with He–Ne laser IR source.

Similarly, phase analyses of the coatings before and after immersion in stimulated body fluid (SBF) were carried out by XRD and FTIR for molecular structural information. Field-emission scanning electron microscope (FESEM) (Zeiss Supra, 35VP, Germany) equipped with energy-disperse spectrometer attachment were utilized to evaluate both top surface and cross-section morphologies and quantitative elemental allocations of the samples before and after immersion. Prior the study, carbon sputter coating was given (∼30 nm) to make the surface conductive. Coating delamination strength was assessed by scratch tester (Scratch Adhesion Tester, Ducom, USA) having diamond indenter Rockwell C, with tip radius 200 μm with increasing load from 1 to 35 N.

#### Electrochemical corrosion test

Electrochemical corrosion of coated and uncoated samples was carried out in SBF solution with an electrochemical workstation (Bio-Logic Science Instruments SAS, France). Setup contains three-electrode cell (kept at 37 ± 0.5 °C) with reference electrode as Saturated calomel electrode (SCE), counter electrode as platinum mesh and sample with 0.64 cm^2^ surface area exposed to the solution as working electrode. Prior the experiment, samples were ground using 1200 grit emery paper, followed by polishing with 1 μm alumina powder and washing with ethanol (99%). The potentiodynamic polarisation data were collected from −0.25 V (with reference to SCE) to 1.6 V with a scanning rate 10 mV/s.

#### SBF immersion test

SBF solution was prepared following Kokubo et al. [37], maintained at 37.4 ± 0.2 °C and pH ~7.4 by tris [tris(hydroxymethyl)aminomethane] buffer during preparation. Surface area of samples to volume of SBF added was maintained at 1 cm^2^/15 mL during the study in closed test tube for 7 and 14 days without replenishing SBF solution in between. Change in weight of sample, pH and supernatant ion concentrations (Mg^2+^, Ca^2+^, PO_4_^3^) was noted with time. After 7 and 14 days, low angle XRD and FTIR were used for assessment of coating composition while FESEM was taken on top surface to verify the microstructure. Quantitative phase analysis of the XRD data was done by using RIR method in X’pert pro HighScore Plus software.

### In vitro biocompatibility assessments

#### Cell culture procedure

Cell culture medium containing DMEM, 10% foetal calf serum and 1% penicillin/streptomycin with human osteoblasts-like cells (MG-63) was used and kept in a humidified atmosphere of 5% CO_2_ at 37 °C. 90% confluence cells were counted following the procedure of trypsinization, centrifugation and finally suspended back in media. Sterilization of the samples was carried out using 70% ethanol and UV light for 30 min followed by washing with sterile PBS (pH 7.4) and treated with DMEM medium for 4 h to generate a conducive atmosphere for better sustaining of cells. Implants were partially dried for 2 h to make certain for superior cell penetration. The next step includes drop-by-drop addition of 20 μL of the cell suspension in medium, containing 10^5^ cells in each sample. The cell loaded samples were kept in a humidified environment, at 37 °C, 5% CO_2_ to facilitate better cell adhesion in the early hour. The cell seeded matrices were kept in medium for 21 days with alternate day replacement of medium.

#### Cell viability assay

MTT assay was carried out to examine the viability of cells at several time points. The procedure involved the incubation of samples in 5 mg/mL MTT [3-(4,5-dimethylthiazol-2-yl)-2,5-diphenyl tetrazolium bromide] stock solution (1:10 dilution) using PBS (pH 7.4). After 4 h of incubation, formazan crystals were dissolved in DMSO and the optical density was measured in spectrophotometer (Bio-Rad, iMark) as per the manufacturer’s guidelines.

#### Cell proliferation by alamar blue (AB) assay

Cell proliferation on matrices was measured using AB dye-reduction assay over 21 days using dye-to-media ratio of 1:10. A microplate reader (Thermo Scientific Multiskan Spectrum, Japan) was used to estimate the dye reduction of the incubated samples at 570 and 600 nm (for 4 h in dark condition) using following equation:

% AB reduction = [(ε_ox_λ_2_)(Aλ_1_) − (ε_ox_λ_1_)(Aλ_2_)/(ε_red_λ_1_)(A′λ_2_) − (ε_red_λ_2_)(A′λ_1_)] × 100…(1) where ελ_1_ (570 nm) and ελ_2_ (600 nm) represent the molar extinction coefficient of AB; ε_ox_ and ε_red_ in oxidised and reduced form, respectively; absorbance of test wells were Aλ_1_ and Aλ_2_ and A′λ_1_ and A′λ_2_ correspond to the absorbance of negative control wells. All given pairs were evaluated at 570 and 600 nm.

#### Alkaline phosphatase (ALP) assay

MG-63 cells (NCCS, Pune, India), seeded with samples, were cultured for a definite time point to quantify the produced alkaline phosphatise spectrophotometrically. Briefly, the cell laden constructs were rinsed with PBS (pH 7.4), homogenised with 1 mL tris buffer (1 M, pH 8.0) and finally sonicated for 4 min in chilled condition. Next, 1 mL of 16 mM p-nitrophenyl phosphate (Sigma) solution was added to 20 µL of the supernatant and incubated for 5 min, at 30 °C. The p-nitrophenol produced in presence of ALP was measured by observing the absorbance at 405 nm depicting absorbance, calculated at 405 nm as p-nitrophenol formed and normalized by incubation duration and cell count: µmole/min/10^5^ cells.

#### Cellular morphology

Orientation, distribution and morphology of cells were monitored by Laser confocal microscopy (Olympus FV 1000, Olympus, Japan). In brief, samples were fixed with 4% paraformaldehyde for 1 h followed by 5 min cellular permeabilization using 0.1% Triton X-100 in bovine serum albumin (BSA). Post blocking of samples with 1% BSA for 1 h, actin filaments were stained using Alexa Fluor^®^ 488 and the nuclei with Hoechst 33342. Later, these were imaged by confocal laser microscopy and analyzed with Olympus FV 1000 Advanced software version 4.1 (Olympus, Japan).

#### Gene expression by real-time RT-PCR

RNA extraction was performed on 21 days cultured MG-63 cells on different samples by using Trizol solution (Invitrogen, USA). In brief, cell seed samples were transferred to small vials containing 1.5 mL of Trizol solution and incubated for 15 min. After that, centrifugation was done at 12000 rpm for 10 min/4 °C and the clear supernatant was collected in a fresh tube followed by addition of chloroform, incubated for 5 min at RT. The sample was then mixed for 15 s and again incubated for 5 min at RT. Again the samples were centrifuged at 12000 rpm for 15 min at 4 °C and the top most aqueous layer was transferred to an RNeasy Plus Mini-Spin Column (Qiagen, Germany). According to the manufacturer’s protocol, the RNA was washed and eluted repeatedly. Then, RNA samples were reverse-transcribed into cDNA using High capacity cDNA reverse transcription kit (Applied Biosystems, USA) in line with the manufacturer’s guidelines.

Using SYBR Green (Applied Biosystems, USA), gene expression was performed by real-time PCR conditions in an ABI Prism^®^ 7000 Sequence Detection System (Applied Biosystems, USA). SYBR Green Supermix, 5 µL cDNA templates and 5 pmol/mL of each primer (forward and reverse) were used for real time analysis in a final solution of 50 µL volume and plates were loaded using a RT loading platform. Cycling conditions involved an initial denaturation step of 8 min and 45 s at 95 °C followed by 45 cycles of 30 s at 95 °C, 30 s at 58 °C and 30 s at 72 °C. Data were collected at 72 °C in each cycle. CT (threshold cycle) values were calculated using the Relative Quantification software (Applied Biosystems).

Highly purified gene-specific primers for osteopontin (OPN), collagen I, osteocalcin (OCN), Runx2 and housekeeping gene GAPDH (Table 2) were selected bearing in mind the literature and synthesized commercially (MWG-Biotech AG Ltd, India). The Ct value of the housekeeping GAPDH gene was used to normalise relative expression levels for each target gene using an identical procedure (2^−ΔΔCt^ formula, Perkin Elmer User Bulletin s ≠ 2). Each sample was experimented in triplicate.

**Table 2:** RT-PCR primer sequences (forward and reverse) used in the current gene expression study

Genes	Forward primer	Reverse primer
Runx2	5′-GCTTCTCCAACCCACGAATG-3′	5′-GAACTGATAGGACGCTGACGA-3′
OCN	5′-AAAGCCCAGCGACTCT-3′	5′-CTAAACGGTGGTGCCATAGAT-3′
Osteonectin	5′-ACAAGCTCCACCTGGACTACA-3′	5′-TCTTCTTCACACGCAGTTT-3′
OPN	5′-GACGGCCGAGGTGATAGCTT-3′	5′-CATGGCTGGTCTTCCCGTTGC-3
ALP	5′-TCAGAAGCTCAACACCAACG -3′	5′-TTGTACGTCTTGGAGAGGGC -3′
BSP	5′-CAGGGAGGCAGTGACTCTTC-3′	5′-AGTGTGGAAAGTGTGGCGTT-3′
COL I	5′-TCCTGCCGATGTCGCTATC-3′	5′-CAAGTTCCGGTGTGACTCGTG-3′
GAPDH	5′-AGGTCGGTGTGAACGGATTTG-3′	5′-TGTAGACCATGTAGTTGAGGTCA-3′

#### Mineralization assay by alizarin red S (ARS) staining

The mineralization study was assessed by using the ARS staining dye. In brief, prior to washing thrice with PBS, MG-63 cells loaded constructs were fixed in ice cold 70% ethanol for 1 h. Next, constructs were again washed with distilled water and stained with ARS (40 mM) for 20 min at RT. At the end, the stained constructs were viewed under optical microscope after rigorous washing with distilled water. The principle of the study is to observe the mineralization which occurs due to binding of ARS with calcium salts. This can be qualified by imaging (mentioned above) and quantified by reading the absorbance at 540 nm in microplate reader prior to desorbing the stain by using 10% cetylpyridinium chloride for 1 h.

### In vivo biocompatibility studies

Nine mature New Zealand white rabbit (1.5–1.8 kg body weight) were utilized in the present in vivo pre-clinical experiment. The rabbits were maintained in separate cages of temperature and humidity controlled room including provision of standard diet and water. The animals were assigned into three random groups consisting of three animals each. In group I (three animals) bare Mg alloy scaffolds (BM) were implanted bilaterally in distal part of femur bone, whereas BMH and BMG implants were placed in other two groups (groups II and III, respectively). Protocol of Institutional Animal Ethical Committee, West Bengal University of Animal and Fishery Sciences (WBUAFS), India, (Approval No. Pharma/188 (viii) dated 31.07.2015) was strictly followed.

### Surgical procedure

Before surgery, distal femur of both hind legs were shaved, cleaned and aseptically prepared. Anaesthesia was achieved with a combination of xylazine hydrochloride (Injection Xylazine, Indian Immunologicals, Ahmadabad, India) at 6 mg/kg body weight and ketamine hydrochloride (Ketalar, Parke-Davis, Hyderabad, India) @33 mg/kg body weight intramuscularly. Skin incision was made in the distal lateral part of the femoral bone in all groups. The femur bone was approached by surgically exposing the skin, subcutaneous tissue, the muscle and finally periosteum. A circular bone defect was created by micromotor dental drilling to press fit the implants. During this procedure, constant sterile cold water was irrigated at the defect site to prevent thermal necrosis of bone. Respective implants were then press fitted and incised muscles, fascia and skin were apposed with standard suturing techniques.

#### Postoperative clinical examinations

During the post-operative period, animals were checked for any lameness, swelling of the operated site, oedema and the cardinal signs of local inflammatory reaction up to 2 months.

#### Radiological examinations

Chronological radiographs of the operated limb were performed at day 0 and afterwards on 1 and 2 months postoperatively (300 mA, M.E. X-Ray Machine, India) to ascertain the proper position of implant within the bony defect.

#### Histological study

Implanted bone area was collected for histology to assess the status of cell–material interaction. Accordingly, implanted bone tissue samples were collected for all the groups on 2 months postoperatively. Bone sections were initially fixed in 10% formalin for 7 days and afterwards decalcified using Goodling and Stewart’s fluid (15 mL formic acid, 5 mL formalin and 80 mL distilled water). The resultant decalcified bone samples were fixed in paraformaldehyde, 4 µm tissue sections was prepared from paraffin embedded block and finally stained with haematoxylin and eosin. The stained tissue sections were finally examined under Leica microscope (Leica Microsystems, Weltzar, Germany) for histological examination.

#### Scanning electron microscopy study

Two month post-implanted bone samples were also checked for interfacial study of new osseous tissue formation using SEM. After carefully removing the soft tissue from the bone, samples were fixed in 2% electron microscope grade glutaraldehyde phosphate solution for 48 h, washed thrice for 30 min with PBS (pH 7.4) and distilled water and finally drying the samples in a series of graded alcohol solutions. Gold sputter coated bone samples were imaged using an FESEM (LEO, UK) for microstructural analysis of newly formed osseous tissue at the interface of bone and material.

#### Fluorochrome labelling study

Oxytetracycline as fluorochrome marker (Terramycin; Pfizer India, India) @25 mg/kg body weight was injected 25 days prior to euthanasia of the animals at 2 months, i.e. on the days 35, 36 and 43, 44 (2-6-2) postoperatively. Retrieved implanted bone samples were ground to 20 µm thickness and finally placed under ultraviolet incidental light with a Leica DM 2000 fluorescence microscope. The golden yellow fluorescence (new bone formation) pixels was measured and consequently converted to percentage of new osseous tissue formation.

#### Toxicological study

Toxicological study of BM, BMH and BMG implanted bone was carried out by histology of three major organs (heart, liver and kidney). To carry out, H&E stained histological sections of heart, liver and kidney from sacrificed animals were prepared to observe any presence of significant cellular changes.

#### Immunocompatibility study

Amount of host-implant immune compatibility was determined by measuring the concentration of IL2, IL6 and TNF-α cytokine response of the animals post in vivo implantation with biomaterials (i.e. BM, BMH and BMG implants). Blood serum was collected at 1, 2, 4 and 8 weeks post surgery and concentrations of IL2, IL6 and TNF-α were estimated using ELISA kits for rabbit (Invitrogen, USA).

### Statistical analysis

Statistical analysis of the in vivo immune response data was carried out using one-way analysis of variance (ANOVA) with a Tukey’s post hoc by ORIGIN software. Absolute mean values and standard deviations were calculated. Data were taken from three samples (*n* = 3).

## Results

### Material characterization

#### Substrate characterization

**Fig. 1:**
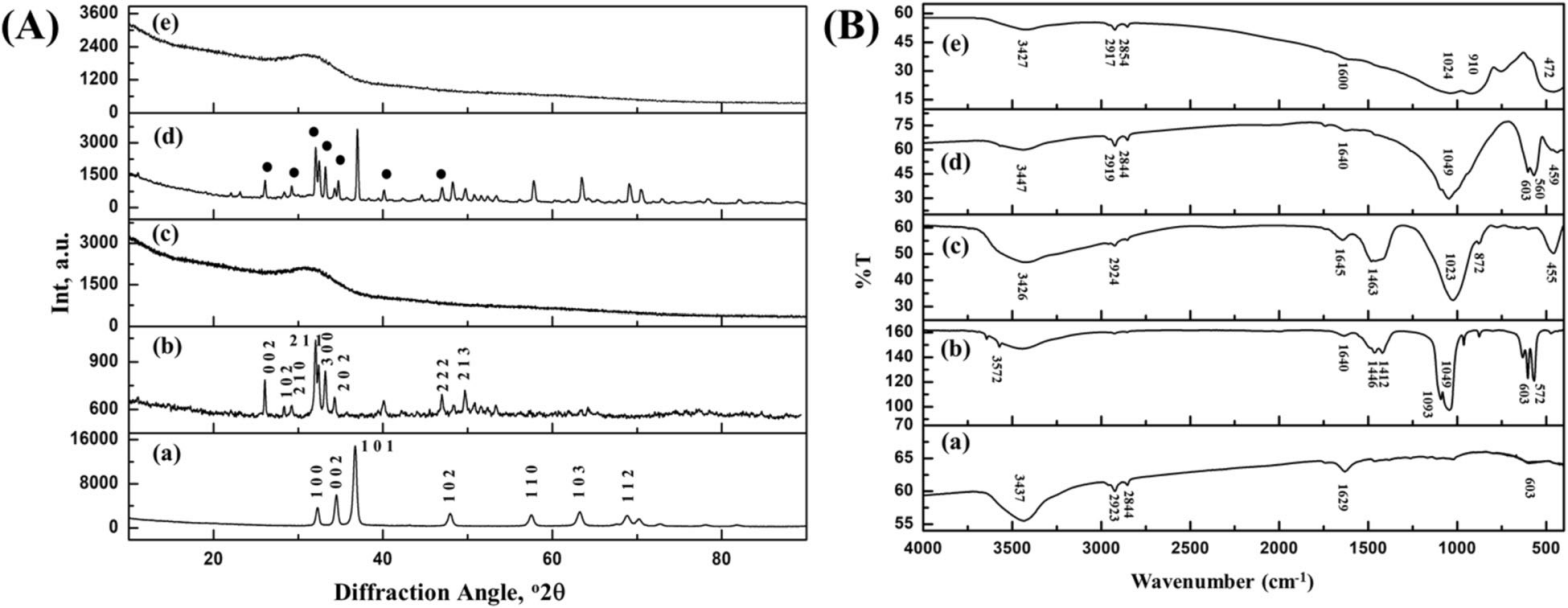
**A** XRD pattern and **B** FTIR spectra of (a) BM, (b) HAp granules fired at 1250 °C, (c) bioactive glass, (d) BMH (•HAp phase) and (e) BMG

**Fig. 2:**
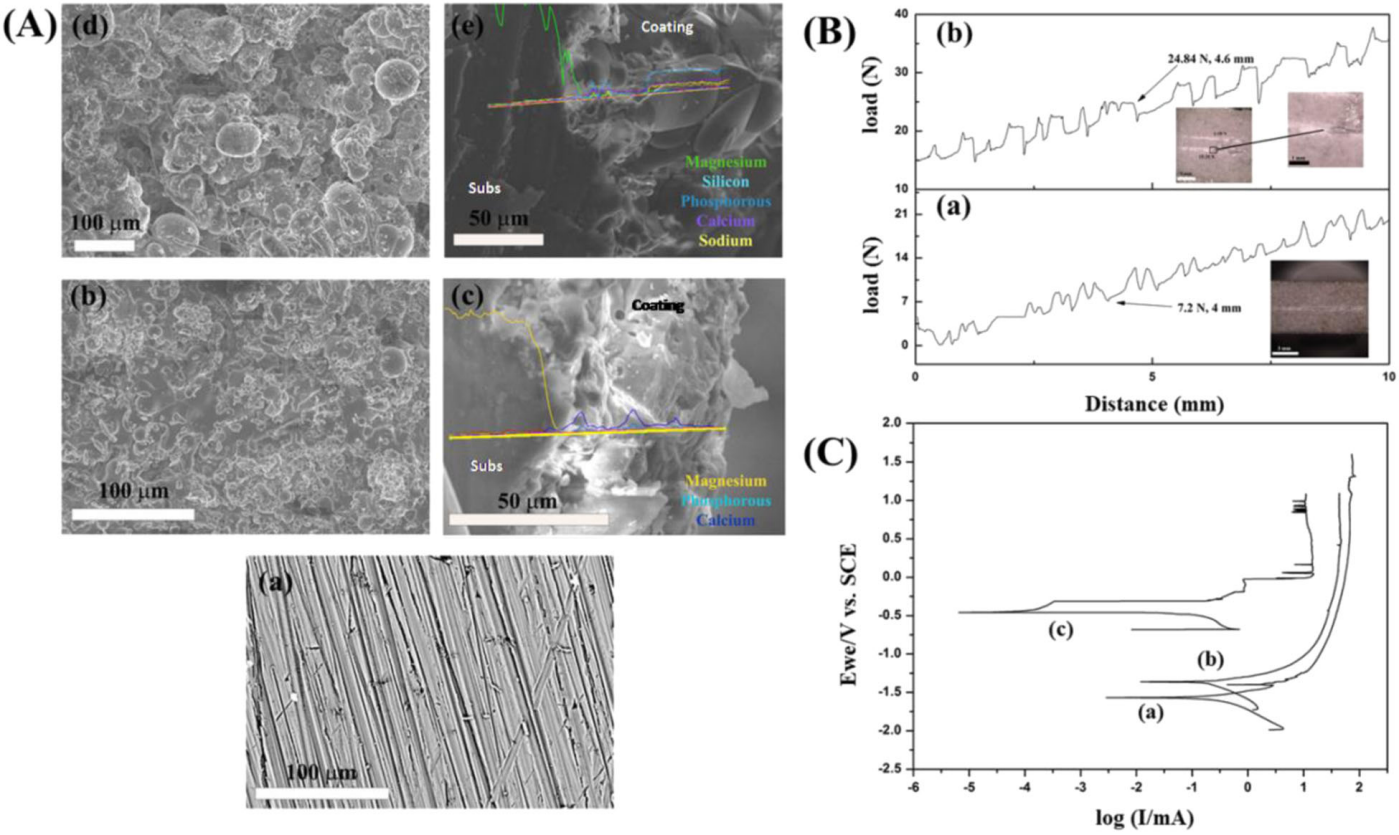
**A** Top surface FESEM [(a), (b) and (d) for BM, BMH and BMG, respectively] and interface FESEM-EDAX [(c) and (e) for BMH and BMG]. **B** Scratch profile of (a) BMH and (b) BMG [inset: optical microscope image of surface]. **C** Tafel plot recorded during corrosion testing in contact with SBF for (a) BM, (b) BMH and (c) BMG samples

XRD, FTIR and SEM-EDX of bare substrate (BM) are shown in Figs. 1A-a, B-a and 2A-a, c, e, respectively. SEM exhibited typical ground surface caused by mechanical grinding having EDX and XRF (not shown) confirming alloying materials Mg, Zn and Ca with impurities (such as SiO_2_, Al_2_O_3_, SrO, etc.). XRD indicated crystalline phase-pure magnesium, matched with JCPDS PDF #00-035-0821, while FTIR supported the presence H_2_O and Mg–O as a result of environmental corrosion.

#### Powder characterization

XRD of graded/sieved (80–120 μm) HAp and BG powders confirmed crystalline HAp phase (Fig. 1A-b) and amorphous glassy phase (Fig. 1A-c) from characteristic peaks [2θ values 31.7°, 32.2° and 32.9° corresponding to (211), (112) and (300) planes and matched with JCPDS PDF# 00-009-0432] and amorphous hump, respectively. FTIR spectra (Fig. 1B-b) showed peaks related to vibrational and stretching of phosphate groups (603, 962 and 1093 cm^−1^) and apatite –OH group (3572 cm^−1^) supporting phase purity of HAp along with peaks corresponding to carbonate group (1640 cm^−1^) as well. FTIR of BG sample (Fig. 1B-c) showed peaks at 1023 cm^−1^ (stretching) and 455 cm^−1^(bending) corresponding to Si–O–Si vibrations and that at 872 cm^−1^ corresponds to O–Si–O stretching. A broad absorption peak at 3426 cm^−1^ corresponds to intermolecular hydrogen bonded OH, whereas presence of molecular water is also evident by sharp peak at 1645 and 2924 cm^−1^and impurities such as ionic nitrates at 1463 cm^−1^. Final composition of BG used for plasma spray coating was (approximately by wt.): 53% SiO_2_, 23% Na_2_O, 20% CaO and 4% P_2_O_5_.

#### Coating characterization

Figure 1A-d, e shows the XRD of coated surface of BMH and BMG samples, respectively. BMH samples showed phase mixture of HAp and Mg (73% and 27%, respectively, calculated from Rietveld analysis). Percentage of crystallinity (calculated by Landi et al.’s method [38]) and average crystallite size (calculated by Scherrer’s method [38]) of HAp phase is found to be about 74% and 19 nm, respectively. Physically, coating coverage was found to be better in case of BMG compared to other samples with no formation of crystalline/other amorphous phase. Amorphicity of S53P4 was increased after plasma spray process which is reflected by lower intensity of XRD pattern.

Figure 1B-d, e shows FTIR spectra of BMH and BMG, respectively. Wider peaks in comparison with spectra of the HAp powder were found in case of BMH samples. Decrease in the crystallinity of BMH (as seen in XRD) has also been reflected in FTIR. No characteristic peaks were found due to carbonate bond as that of base HAp powder. Broadening of peaks at 560, 603 and 1049 cm^−1^ and decrease in intensity at 3752 and 632 cm^−1^ can be observed in case of BMH samples. Peak broadening (at 472, 1024 and 1600 cm^−1^) can be seen in case of BMG sample also after plasma spray coating.

Figures 2A-b, c and A-d, e show FESEM microstructures of top and interface of plasma sprayed BMH and BMG, respectively. At interface, layer-wise fish scale-like morphology of melted and deformed splat can be seen near vicinity of substrate and at top globular shaped splat along with some porosity (~1–10 μm) were seen around outside/periphery of BMH samples together with some unmelted particles. Dimensions of pores for BMG were slightly higher (15–30 μm) with layer-wise globular shaped unmelted/unreacted particles. The thickness of the coating was found to be 50–60 μm for BMH and 90–100 μm for BMG samples. There was sharp decrease in Mg concentration which can be seen from EDAX line scan across interface (cf. Fig. 2A-c, e).

Load-displacement plot obtained after scratch test on BMH and BMG are shown in Figs. 2B-a and B-b, respectively; corresponding optical microscope images of the scratch are also provided. From these results, the delamination load was found to be 7.2 N at 3.8 mm for BMH, whereas in case of BMG, it was 24.84 N at 4.8 mm.

##### Electrochemical properties

Potentiodynamic polarization curves for BM, BMH and BMG samples are shown in Fig. 2C-a–c, respectively. Corresponding data of corrosion potential (*E*_corr_) and corrosion current density (*i*_corr_) were evaluated from the curves. For BM, BMH and BMG, *E*_corr_ (mV) values were −1540, −1420 and −296, respectively, with corresponding *I*_corr_ (μA) values as 250, 297 and 68, respectively. Decrease in *E*_corr_ of BMH and BMG clearly indicate that both coatings have relatively better resistance to corrosion initiation than BM. However, the corrosion rate appears to be lowest for BMG due to their lowest *I*_corr_.

##### SBF immersion test

**Fig. 3:**
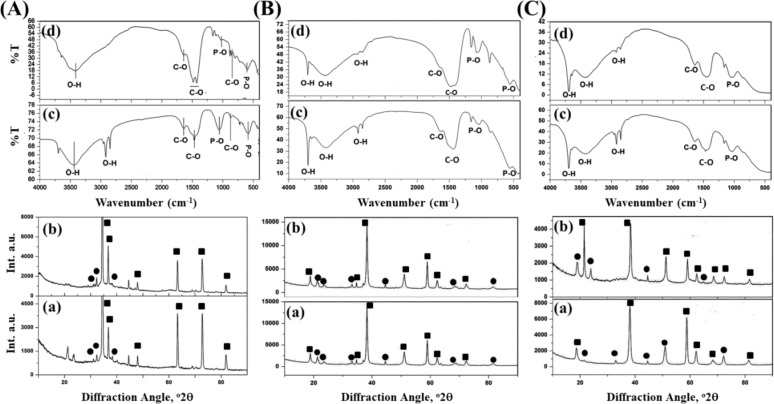
XRD pattern and FTIR spectra of the samples after 7 and 14 days of SBF test. **A** BM. **B** BMH. **C** BMG. (a) and (b) are XRDs and (c) and (d) are FTIRs after 7 and 14 days, respectively, [• HAp phase and ■ Mg phase]

Figure 3A-a, b, B-a, b and C-a, b shows the XRD pattern after 7 and 14 days of SBF immersion test of BM, BMH and BMG samples, respectively. After 7 days of immersion, the XRD of BM showed 11% HAp (JCPDS PDF #00-19-0272) along with 89% Mg (JCPDS PDF #00-035-0821). Mg phase was found to increase up to 92.7% after day 14 confirming corrosion on the surface. XRD of BM samples also showed decrease in HAp phase crystallinity (11–7.3%) as well as average crystallite size (83–33 nm) after day 14. Both BMH and BMG samples showed formation of HAp (JCPDS PDF #01-086-1201) and calcium phosphate hydroxide (JCPDS PDF #01-083-1887) along with Mg(OH)_2_ (JCPDS PDF #01-076-0667 and #00-044-1482, respectively). Percentage of phases in case of BMH samples was calculated using X’pert pro software and was found to be 7.5% HAp (with average crystallite size 11 nm) and 92.5% magnesium hydroxide. XRD taken after 14 days showed decrease in percentage (7.5–4.5%) of HAp phase (with average crystallite size 4.7 nm). However, in case of BMG samples, Ca–P phase was found to increase from day 7 (31.3%) to day 14 (67.3%) along with average crystallite size of HAp (9.3–11 nm).

FTIR spectra of BM, BMH and BMG samples after days 7 and 14 of SBF immersion study, as shown in Fig. 3A-c, d, B-c, d and C-c, d, supported XRD findings. BM samples showed peaks related to carbonated apatite at 563, 872, 1054, 1468 and 3435 cm^−1^. Based on FTIR result, after SBF study of BM samples, the layer obtained contains phosphates and carbonates. A sharp P–O bending mode doublet at 592 cm^−1^ is suggestive of HAp [39]. However, decrease in C–O and P–O peak intensity supports the decrease in crystallinity as stated in XRD. BMH and BMG samples, on the other hand, showed fingerprints of apatite phase (563, 872, 1042, 1166, 1424, 1640, 2924 and 3700 cm^−1^ in case of BMH samples and 872, 1042, 1468, 1640, 2924 and 3696 cm^−1^ in case of BMG samples) along with indication of Mg–O bonding at 450–500 cm^−1^ wavenumber. A detailed band interpretation is given in Table 3. BMG samples showed more apatite formation than BMH samples after SBF immersion study.

**Table 3:** FTIR peak analysis after SBF immersion study

	BM (cm^−1^)		BMH (cm^−1^)		BMG (cm^−1^)	
	Day 7	Day 14	Day 7	Day 14	Day 7	Day 14
Mg–O	424	420	424	424	424	424
P–O			563	563		
P–O	585	592				
C–O/HPO_4_^3−^	872	879	872	872		872
P–O			1042		1042	1042
P–O	1054			1054		
P–O		1166	1166	1166	1166	1166
C–O		1424	1424	1424		
C–O	1468	1476			1468	1468
H–O–H	1640	1640	1640	1640	1640	1640
Absorbed H_2_O	2851		2851	2851	2851	2851
Absorbed H_2_O	2924		2924	2924	2924	2924
H–O–H	3435	3426	3427	3427	3427	3427
O–H	3700	3694	3700	3700	3700	3696

**Fig. 4:**
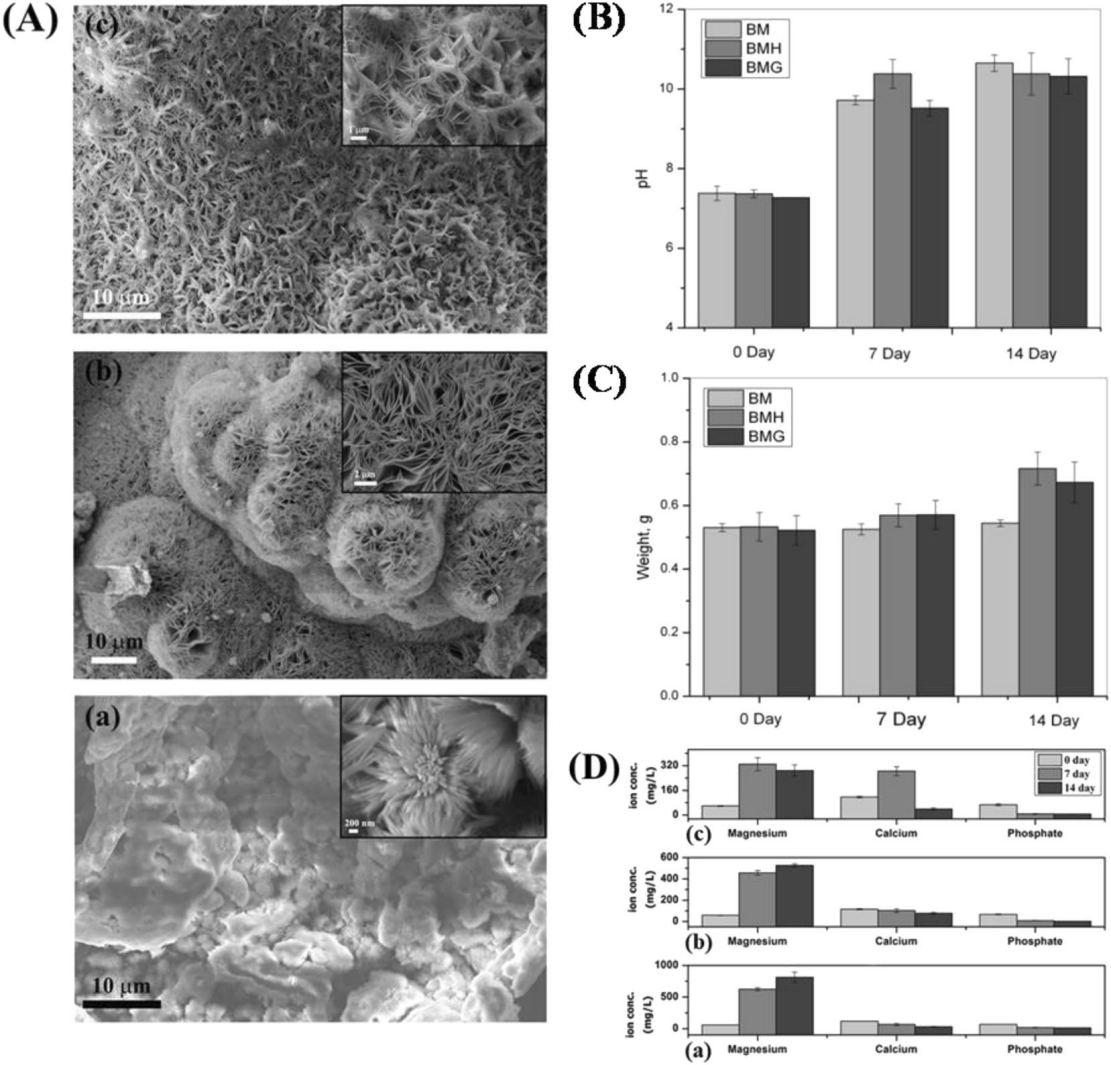
**A** FESEM microstructures after 14 days of SBF study; change of **B** pH, **C** weight and **D** magnesium, calcium and phosphate ion concentration of supernatant of different samples at days 0, 7 and 14. (a) BM, (b) BMH and (c) BMG

After SBF immersion for 14 days, top surface of bare and coated Mg alloy substrates were found to have different morphologies due to interaction with SBF. BM samples showed (Fig. 4A-a) flower-like apatite deposition, primarily composed of needle shaped crystals covering the entire surface. BMH samples (Fig. 4A-b) exhibited globular apatite microstructure composed of fine interconnected flakes with pores (0.1–1.5 μm). There was layer-wise apatite formation and when observed at higher magnification the precipitates revealed flake-like crystals with small pores in case of BMG (0.5–1.5 μm) (Fig. 4A-c and inset) and morphology was found to be denser and closely packed than others.

pH of supernatant solution collected from BM, BMH and BMG samples increased with time (Fig. 4B) in varying rates. Though, after initial 7 days, the increase in pH was higher for BMH samples, however, BMG samples was found to be higher after day 14. Qualitatively, the change in weight of the samples (Fig. 4C) was found to be directly proportional to the formation of apatite on these surfaces. Negligible weight change of BM samples indicate its inability towards apatite formation, whereas the weight grain of BMH (average increase after 7 day was ~3.4% and after 14 day it was ~12.9%) and BMG (average increase in weight after 7 day was ~8.7%, after 14 days this was ~27.2%) demonstrate their apatite precipitation ability. Changes in ion concentration of supernatant with immersion time are another way of correlating bioactivity (w.r.t. apatite formation) as well as corrosion (Fig. 4D-a–c). BM showed increase of Mg ion concentration with time, whereas the solution of BMH samples showed initial increase of Mg ions up to day 7 but the rate was decreased at day 14. In case of BMG samples, however, Mg ion was found to be decreased after day 7–14. Calcium ion concentration was decreased in case of BM and BMH after days 7 and 14, but the same increased in BMG after day 7 and eventually deceased at day 14. Supernatant corresponding to BMH and BMG showed lower concentration of phosphates which support higher bioactivity (w.r.t. apatite formation) of the samples.

### In vitro biocompatibility assessments

**Fig. 5:**
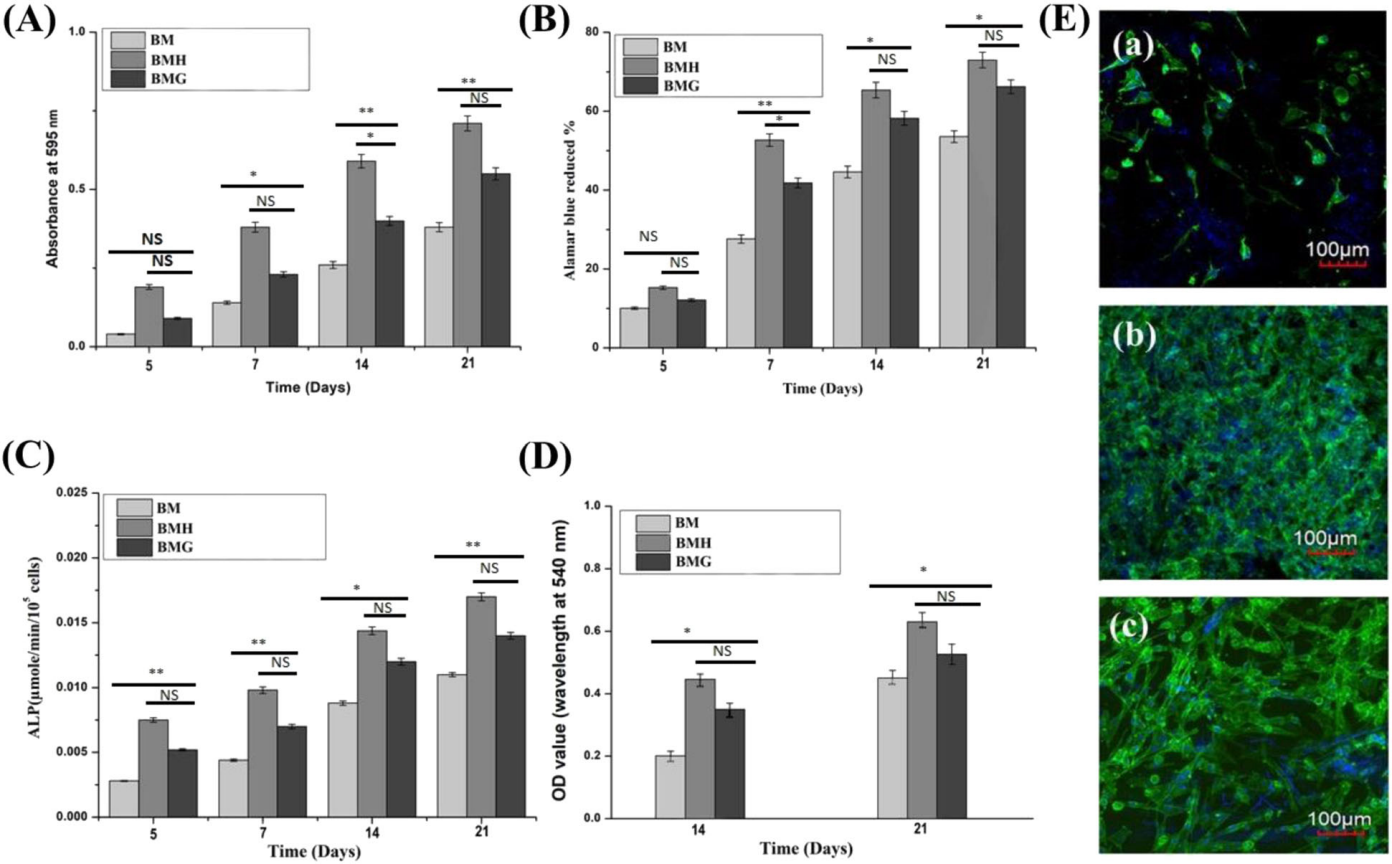
MG-63 **A** Cell viability, **B** proliferation, **C** ALP expression and **D** mineralization assay of different samples. **E** Cell morphology (by confocal laser microscopy) of (a) BM, (b) BMH and (c) BMG samples

MTT and AB assays were conducted to assess the cytotoxicity of samples. Both results showed that these implants are non-toxic and provide favourable surfaces for cellular proliferation. MG-63 cell viability, cell proliferation and ALP expression on BM, BMH and BMG samples were measured on days 5, 7, 14 and 21 days. Maximum cell viability (**p < 0.01, ***p < 0.001), proliferation (*p < 0.05, ***p < 0.001) and ALP expression (**p < 0.01, ***p < 0.001) were recorded for BMH after 21 days of cell culture as shown in Fig. 5A–C, respectively. Calcium (Ca^2+^) deposition, on the other hand, measured on day 14 and 21 days (Fig. 5D) showed maximum for BMH again after 21 days of cell culture (**p < 0.01, ***p < 0.001).

#### Cell morphology

Laser confocal helped to examine cell morphology and spreading of cells on samples. Cell morphology on BM, BMH and BMG are presented in Fig. 5E-a–c. Maximum number of cells are present in BMH as compared to other coated samples. Actin covers total surface of this sample and formed neo matrix which penetrated to sample as well. Actin filaments were stained with Alexa Fluor^®^ 488 (green), nuclei with Hoechst 33342 (blue) and finally examined under confocal at 20×.

Cell growth upon different layers of 3D constructs was observed by Z-scanning during confocal microscopy and finally image was taken by merging the different layers. The image depicted abundance and homogeneously dispersed actin filaments on BMH and BMG samples compared to BM. BMH samples showed presence of abundant cells followed by BMG. However, actin sharing was meagre and secluded for BM to just around the cell nuclei.

**Fig. 6:**
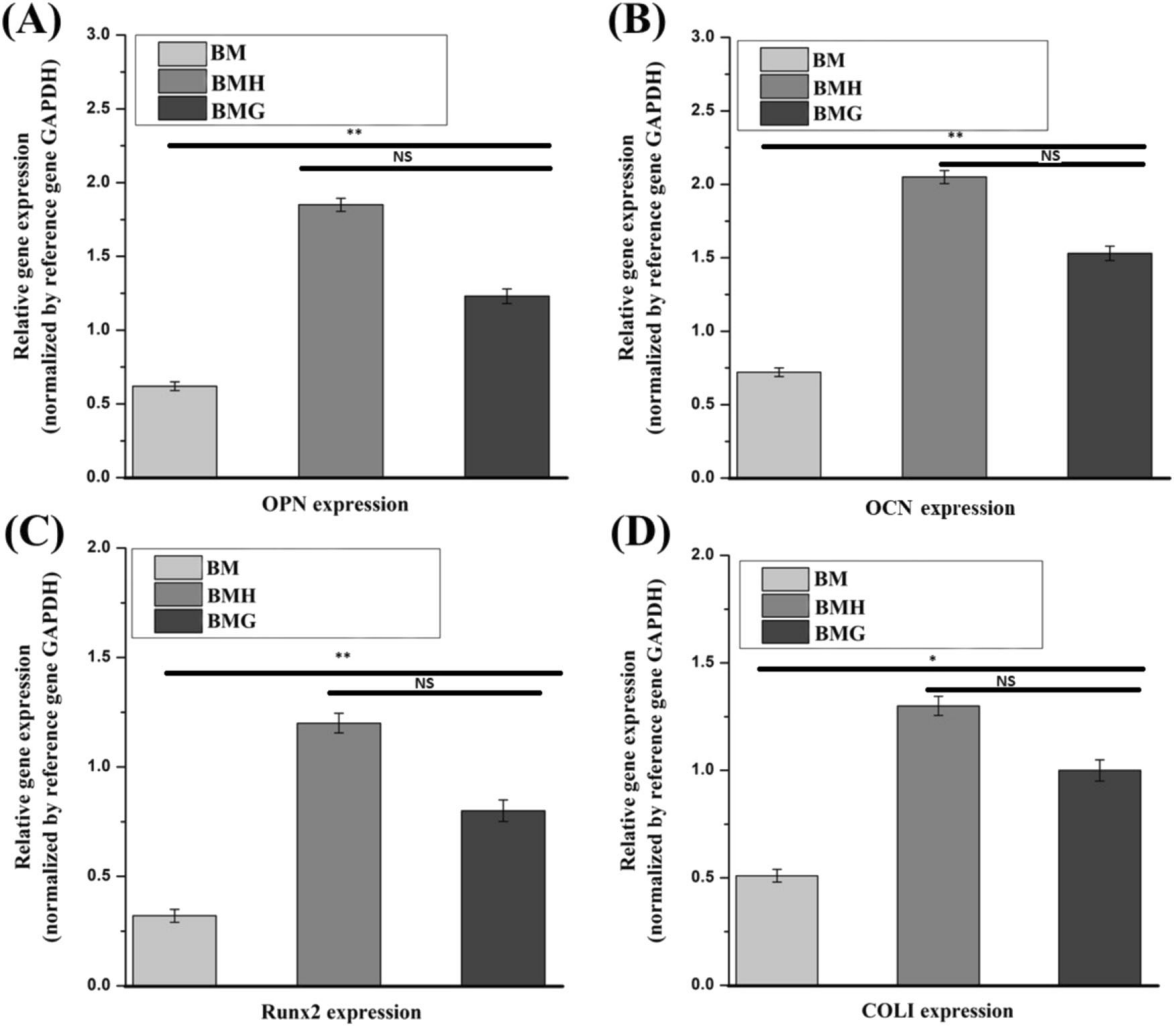
Relative gene expressions (normalized by reference gene GAPDH) w.r.t. **A** OPN, **B** OCN, **C** Runx2 and **D** COLI expression of different samples

mRNA expression of representative bone-associated genes, such as OPN, collagen I, OCN and Runx2, help to investigate osteogenic efficacy of different implants. Figure 6 shows the comparison of the gene expression of cells on various samples after 21 days of culture. BMH and BMG samples exhibited relatively higher levels of genes compared to BM samples. However, after the study, substantial difference in gene expression between BMH and BMG was not observed.

### In vivo studies

#### Bone histology

**Fig. 7:**
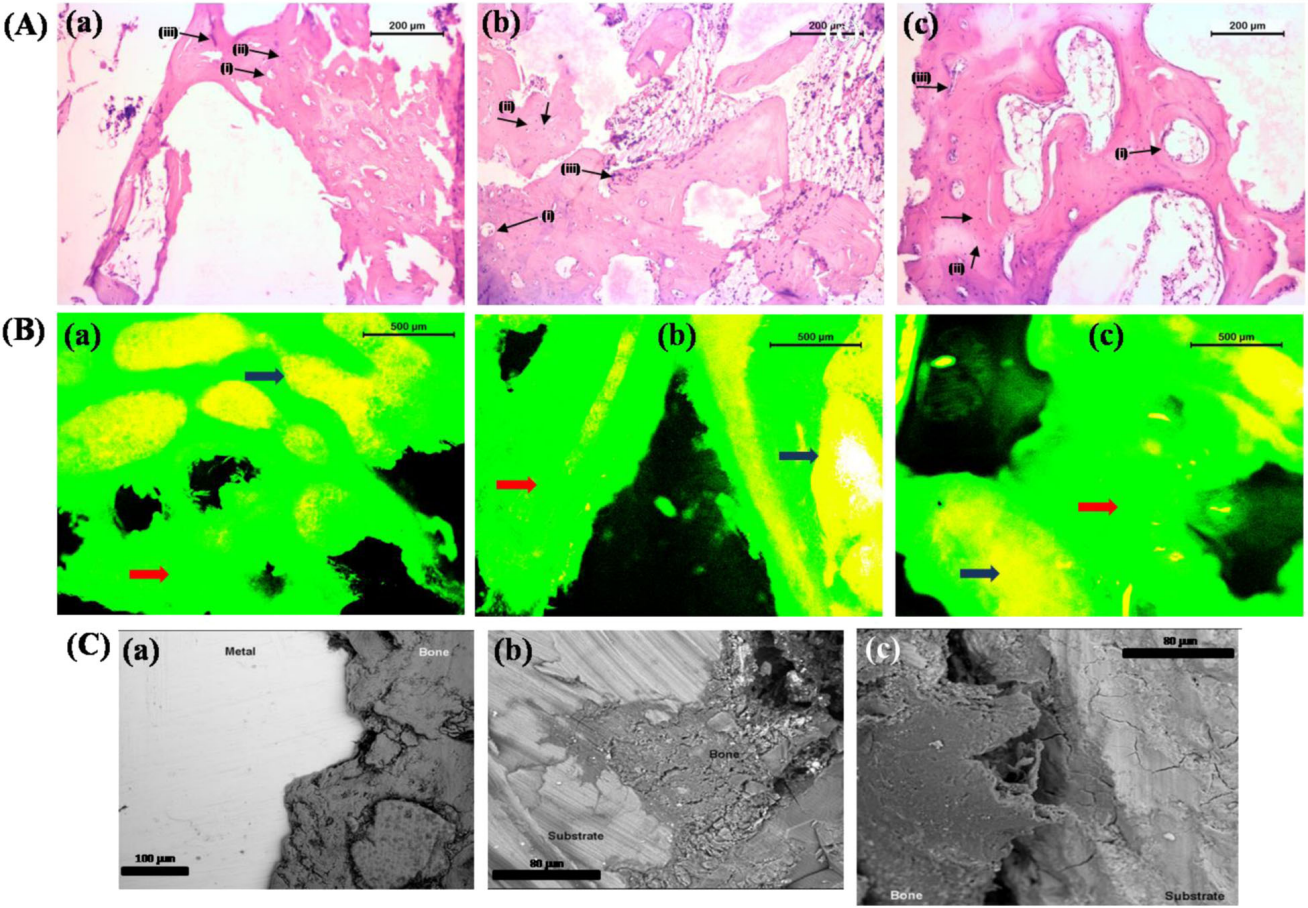
**A** Histology [(i) haversian canal, (ii) osteoblasts, (iii) osteoclasts]. **B** Fluorochrome labelling [blue arrow—new bone formation; red arrow—old bone]. **C** SEM images of implanted bone samples after 2 months [(a) for BM, (b) for BMH and (c) for BMG]

Figure 7A-a–c shows histological picture of implanted bone at 2 months. As shown in Fig. 7A-a, BM implanted bone section depicted a well-developed bony matrix with sufficient number of Haversian canal, bony lacunae and few osteoclasts. Medullary portion was less avascular and occupied by few osteoclasts, osteocytes and scanty amount of mucin. Accumulation of osteocyte was prominent in cortical area. Angiogenesis towards medullary region was lesser in amount (Fig. 7A-a). Bony section of BMH implant depicted bony lamellae characterized by well-developed Haversian system, canaliculi and resorption of bone in pericortical areas. Medullary region was occupied by RBC, scanty amount of mucin, few osteoblasts and numerous osteocytes. Bony lacunae in some places were invaded by few osteoclast cells. Angiogenesis was prominent in medullary portion although fibrovascularisation was sufficient in cortical mass (Fig. 7A-b). Figure 7A-c shows histological images of BMG implanted bone section. Section depicted presence of abundant osteocytes, osteoclasts and osteoblast. Fibrovascularisation was prominent in cortical area and perimedular area. Medullary cavity had adipose tissue, few RBC, moderate amount of mucin and osteoblast cell. Angiogenesis was fewer in cortical area than in medullary area (Fig. 7A-c).

#### Fluorochrome labelling study

Golden yellow florescence in the section depicted new osseous tissue formation, whereas dark sea green colour designates host bone. The BM implant samples at 2 months showed new bone formation mainly at centre part and partially in edges, as pointed out by the presence of golden yellow fluorescence (Fig. 7B). New bone formation was nearly 23% in defect area. BMH implant group depicted ample new osseous tissues (~37%) in contrast to other two groups. Although golden yellow fluorescence was mostly limited to peripheral side, a wide deep area of new bone formation was observed. Along this deep zone of golden fluorescence, a narrow band of fluorescence throughout the length was also observed nearly to central part of section indicating bone formation both in central as well as in peripheral zone. In BMG implant group, new bone formation was nearly 29%, mostly in periphery. The new osseous tissue was measured using ImageJ software. The golden colour pixels were calculated and changed to percentage using scale bars from three images each.

#### SEM of bone–implant interface

Scanning electron microscopy of bone–implant interface of BM, BMH and BMG samples are given in (Fig. 7C). It was found that BMH showed best bone apposition in due course of time with little or no interfacial gap, while BMG had also shown similar trend, but due to its conversion of apatite-like layer, metal surface was found to be covered by apatite-like layer with an apposition of bony soft tissue at interface. On the other hand, BM was found to be replaced with associated interfacial bone with time. Matured bony tissues were noticed in case of BMH with time.

#### Radiology

**Fig. 8:**
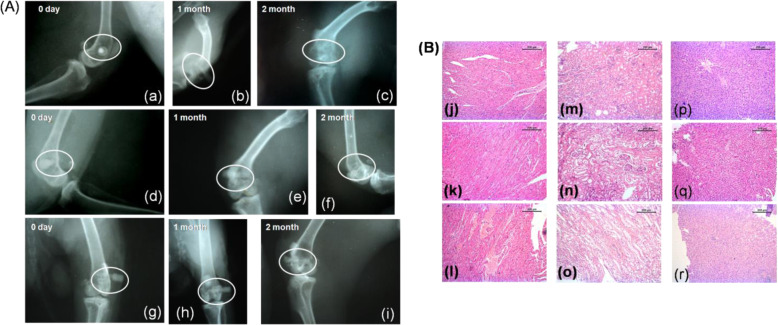
**A** Radiographs of BM (a–c), BMH (d–f) and BMG (g–i) implanted bone immediately after implantation (day ‘0’), 1 month and 2 months post surgery. **B** Histological images of heart (j–l), kidney (m–o) and liver (p–r) of BM, BMH and BMG implanted at 2 months post surgery

Figure 8A shows sequential radiology of different implants in distal metaphysis of femur bone in rabbit model. In BM implant, day ‘0’ (day of implantation) radiographs showed a radiodense circular material placed in metaphysis of the distal femur. After 1 month, the material radiodensity was reduced in comparison to earlier time point. A negligible impression of implant was found after 2 months indicating maximum degradation of material vis-à-vis moderate osseous growth in the defect site. Radiodensity of material and bone is comparable (Fig. 8A-a–c). In BMH implant, ‘0’ day radiograph showed radio-opaque material in the distal femoral bone defect. By the end of 1 month, implant was visible in the defect region with comparable radiodensity of host bone although there was a distinct radiolucent gap between bone and implant. At 2 months, implant was visible with similar radiodensity of host bone but there was reduction in diameter of implant indicating degradation was under process. Radiolucent gap between implant and bone was reduced indicating new osseous tissue formation from the host bone (Fig. 8A-d–f). In BMG implant, ‘0’ day radiograph showed presence of radiopaque implant in created defect of distal metaphysis of femur bone extending up to opposite cortex of bone. At 1 month, implant was visible with comparable radiodensity of host bone. Implant within the bone was under process of degradation as observed by reducing diameter of implant. Radiolucent gap in between implant and bone is negligible. The implant at 2 months showed moderate degradation as observed with loss of round shape of diameter within bony cavity. Radiodensity of implant is approaching to bone density and new bony tissue ingrowths over the defect area (Fig. 8A-g–i).

#### Toxicity study of vital organs

##### Heart

Figure 8B-a–c shows histological section at 2 months of implantation. Architectural detail of BM implanted heart myofibril retained its vitality with all processes including nuclear prominence, intact cytoplasm and fibrovascular network. Vascularisation of total structure was quite normal. Cellular details and infiltrating cells were within normal limit (Fig. 8B-a). In BMH implant group, section depicted almost normal structure of myocardial tissue characterized by well nuclear detail, cytoplasmic organelles and regularly arranged fibres. Mononuclear cells were predominant in some places without involving oedema or other exudation (Fig. 8B-b). In BMG implant group, section depicted a normal architectural pattern of cardiac tissue without any presence of infiltrating cells (Fig. 8B-c).

##### Kidney

In case of BM implant, kidney section depicted normal glomerular tufts, tubular architecture and well-maintained collecting ducts. Peri-glomerular spaces showed normal architectural detail with mild infiltrating cells. Few tubular lining epitheliums showed degeneration to some degree but within normal limit (Fig. 8B-d). Renal architecture of BMH implant was quite normal with glomerular tufts formation and different intact renal tubules. Oozing of RBCs in inter-tubular spaces was seen focally and some tubular epithelium showed necrosis and infiltration with mononuclear cells (Fig. 8B-e). In BMG implant, section showed normal architecture of kidney with glomerular tufts formation and different intact renal tubules (Fig. 8B-f).

##### Liver

Section of BM implanted liver depicted normal limits of hepatocytes. Some portion of total portal triads showed few infiltration and RBC extravasations. Few hepatocytes showed focal changes with mild necrosis (Fig. 8B-g). In BMH, section depicted normal hepatic architectural detail characterized by well-formed hepatocytes, portal triads and well-made globules. Infiltration of mononuclear cells and von Kupffer cells were within normal limit (Fig. 8B-h). In BMG implant group, hepatic parenchyma showed presence of RBC, mononuclear cell, well-formed central veins and few von Kupffer cells (Fig. 8B-i).

#### In vivo immune response

**Fig. 9:**
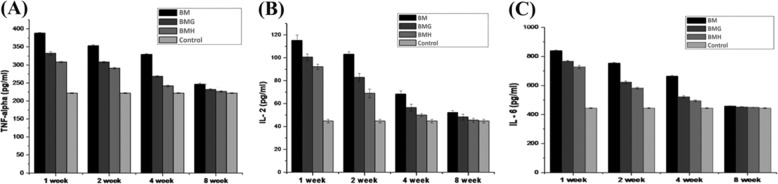
Expressions of **A** TNF-α, **B** IL-2 and **C** IL-6 at 1, 2, 4 and 8 weeks for different samples with the reference of control (normal healthy animals)

Figure 9A–C shows the expression of TNF-α, IL-2 and IL-6 at 1, 2, 4 and 8 weeks. The expression of IL-2 at 1, 2, 4 and 8 weeks showed that at the 1st week, the inflammatory response due to the materials increased maximally followed by a gradual fall at the 2nd, 3rd, 4th and finally at the end of 8th week where the immunoreactivity of the implanted animals returned to its baseline as compared to control (normal healthy) animals. **p < 0.01 and *p < 0.05, n = 3 at each time point (one way ANOVA). The expression of IL-6 at 1, 2, 4 and 8 weeks showed that at the 1st week, the inflammatory response due to the materials increased maximally followed by a gradual fall at the 2nd, 3rd, 4th and finally at the end of 8th week where the immunoreactivity of the implanted animals returned to its baseline as compared to control (normal healthy) animals. ***p < 0.001, **p < 0.01 and *p < 0.05, n = 3 at each time point (one way ANOVA). The expression of TNF-alpha cytokine response at 1, 2, 4 and 8 weeks showed that at the 1st week, the inflammatory response due to the materials increased maximally followed by a gradual fall at the 2nd, 3rd, 4th and finally at the end of 8th week where the immunoreactivity of the implanted animals returned to its baseline as compared to control (normal healthy) animals. **p < 0.01 and ***p < 0.001, n = 3 at each time point (one way ANOVA).

## Discussion

Magnesium ion has different role in numerous biological functions, for instance, in bone. Bivalent magnesium ions help formation of biological apatite as well as have stimulatory effect on growth of marrow cells [40]. It was also reported that adding up of magnesium in nutrients helps in bone metabolism, whereas deficiency leads to lowered bone escalation and amplified bone resorption [41]. Now, in case of Mg alloy implants, depending on its composition, faster degradation is observed within bony environment especially in cancellous part of bone than cortical part [3]. Due to rapid degradation of bare Mg-based implants, attempts have also been made to trigger the mechanical integrity irrespective of the implantation site.

Mg alloy mimics comparable elastic modulus and mechanical strength as cortical bone leading to bone regeneration [42]. In physiological environment, Mg alloy decays in contact with water (body fluid) as shown below. Degradation of Mg alloy in body fluids produces Mg^2+^ cations which are efficiently excreted by kidneys and eliminate them naturally through urine

$${\mathrm{Mg}}\,\left( s \right) + 2{\mathrm{H}}_2{\mathrm{O}}\,\left( {\mathrm{l}} \right) \to {\mathrm{Mg}}^{2 + } + 2{\mathrm{OH}}^ - + {\mathrm{H}}_2\left( {\mathrm{g}} \right)$$

$${\mathrm{Mg}}^{2 + } + 2{\mathrm{OH}}^ - \rightleftharpoons {\mathrm{Mg}}\left( {{\mathrm{OH}}} \right)_2\left( {\mathrm{s}} \right)$$

Rapid degradation of Mg and its alloys is a serious concern for degradable implant application. Therefore, several surface modification approaches have been attempted by different groups [43]. Surface modification not only ensures better mechanical integrity by providing resistance against corrosion in biological fluids but also improves bioactivity of these alloys depending on type of coating material. For temporary fracture fixation devices, this combined advantage was found to be suitable, because this could alleviate necessity of second surgery for removal of implant thereby reducing hospital cost and surgical complications too [44]. Among various surface modification techniques, plasma spraying technique helps in chemical control, bio-corrosion resistance as well as reduced substrate fatigue resistance. Further, plasma spraying process enhances surface properties and biocompatibility keeping excellent bulk properties unchanged. In particular, the technique has many advantages in biomedical applications, for example, with regard to film chemistry, better coating adhesion, conformal and pin-hole free films and enhanced infiltration [45]. In the present investigation, plasma spraying was chosen in order to get a firm adhesion/layer-wise formation of coating with porosity which would act as nucleation site for apatite formation and help different cell functions, ultimately increasing the biocompatibility of the sample. For the first time, plasma spray method using BG material has been employed for a new Mg alloy composition. In plasma spray process using HAp, powders experience high flame temperature, which causes evaporation of water (trapped within pores or part of the HAp lattice structure), resulting decrease in crystallinity (Fig. 1A-d) and low adhesion strength (Fig. 2B-a) [46]. Amorphous S53P4 powders showed firm apposition with the substrate surface after plasma spray coating [which reflected in better adhesions strength (Fig. 2B-b)] without any trace of magnesium phase in XRD (Fig. 1A-e). Microstructure of BMH showed melted and deformed splat near to its substrate, whereas globular shaped splat at the periphery of coating surface (Fig. 2A-c), which can be attributed to better surface cooling on outer surface as compared to inner layers; more cooling led them to more thermodynamically stable shape. Due to its amorphous nature, BAG forms less irregular and globular shaped splat on the surface (Fig. 2A-d). Pores having size ranging 1–10 μm were seen in case of BMH samples, whereas BMG showed interconnected pores ranging 15–30 μm. Interface study confirmed presence of pores up to layers adjacent to BMH substrate which explains lower delamination strength as well as availability of Mg phase in XRD as an effect of Mg ion migration from substrate, on the other hand amorphous BMG showed absence of pores even up to layers adjacent to substrate confirming firm layer-wise formation of coating on surface of substrate. Better homogeneity and superior coverage of BG coating (for BMG) ensured increase in resistance towards corrosion when immersed in SBF solution showing a very high *E*_corr_ value (−296.246 mV vs. SCE) compared to BM (−1540.824 mV vs. SCE) and BMH (−1420.479 mV vs. SCE) samples. Lower resistance of BMH was due to surface pores, which act as pitting corrosion sites [47]. Corrosion current densities (*i*_corr_) of coated and uncoated samples indicated that BMG samples exhibited lowest thermodynamic tendency to participate in anodic reaction thus effectively improving resistance followed by BMH and BM samples [48]. However, after initial corrosion, BMH samples tends to form inactive layer of Mg(OH)_2_, which reduced surface reactivity towards corrosion as stand-alone coating [32]. When immersed in ionic solutions which consist of chloride (Cl^−^) ion (SBF), this passive layer converts from Mg(OH)_2_ to soluble MgCl_2_with time, weakening the surface as well as releasing OH^−^ ions in solution increasing the pH (Fig. 4B). MgCl_2_ dissolves easily in SBF and releases Cl^−^ to continue the chain of corrosion on surface

$${\mathrm{Mg}}\left( {{\mathrm{OH}}} \right)_2 + {\mathrm{Cl}}^ - = {\mathrm{MgCl}}_2 + {\mathrm{OH}}^ -$$

$${\mathrm{MgCl}}_2 = {\mathrm{Mg}}^{2 + } + {\mathrm{Cl}}^ -$$

SBF immersion study, which also involves corrosion, is a very complicated phenomenon where ion exchange occurs simultaneously for apatite formation as well as corrosion. When immersed in SBF, leaching of ions from substrate to solution occurs initially followed by apatite precipitation. Dissolution of calcium ion in case of BMH and BMG control formation of apatite phases on surface. Ca^2+^ ions of the coatings and ions from SBF solution underwent reduction reaction simultaneously with conversion of Mg to MgCl_2_ releasing OH^−^ in the solution, which increases the pH as well. Essentially, the reaction stages are outlined by the following equations

**Table:** 

${\mathrm{Mg}} = {\mathrm{Mg}}^{2 + } + 2{\mathrm{e}}$	$2{\mathrm{H}}_2{\mathrm{PO}}_4^ - + 2{\mathrm{e}} \to 2{\mathrm{H}}_2{\mathrm{PO}}_4^{2 - } + {\mathrm{H}}_2$
$2{\mathrm{H}}_2{\mathrm{O}} + 2{\mathrm{e}} = {\mathrm{H}}_2 + 2{\mathrm{OH}}^ -$	$2{\mathrm{H}}_2{\mathrm{PO}}_4^{2 - } + 2{\mathrm{e}} \to 2{\mathrm{PO}}_4^{3 - } + {\mathrm{H}}_2$
${\mathrm{Mg}}^{2 + } + 2{\mathrm{OH}} = {\mathrm{Mg}}\left( {{\mathrm{OH}}} \right)_2$	$10{\mathrm{Ca}}^{2 + } + 6{\mathrm{PO}}_4^{3 - } + 2{\mathrm{OH}}^ - \to {\mathrm{Ca}}_{10}\left( {{\mathrm{PO}}_4} \right)_6\left( {{\mathrm{OH}}} \right)_2\left( {{\mathrm{BMH}}} \right)$
${\mathrm{Mg}}\left( {{\mathrm{OH}}} \right)_2 + {\mathrm{Cl}}^ - = {\mathrm{MgCl}}_2 + {\mathrm{OH}}^ -$	$5{\mathrm{Ca}}^{2 + } + 3{\mathrm{PO}}_4^{3 - } + {\mathrm{OH}}^ - \to {\mathrm{Ca}}_5\left( {{\mathrm{PO}}_4} \right)_3\left( {{\mathrm{OH}}} \right)\,\left( {{\mathrm{BMG}}} \right)$

**Fig. 10:**
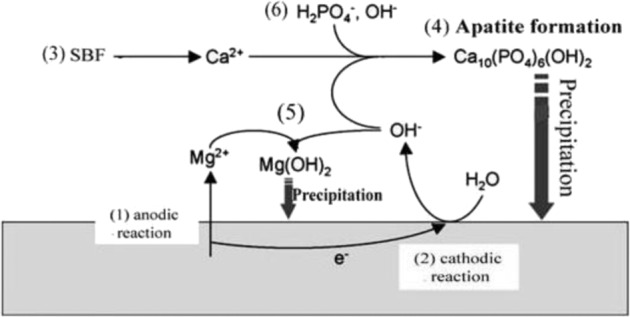
Proposed mechanism of corrosion and apatite formation when immersed in SBF

Hence, more corrosion leads to higher pH as well as higher Mg^2+^ concentration after day 7 in case of BMH samples where porosities in the coating most probably let SBF penetrate to Mg interface which gradually decreases with increasing apatite precipitation up to day 14 with increasing final weight as well. On the other hand, slow increase in pH from day 0 to 7 until day 14 illustrated slower corrosion rate of BMG samples. Therefore, formation and crystallization of apatite layer retards aggressive corrosion during initial days. High calcium concentration of SBF related to BMG samples after day 7 supports leaching of BG which gradually react with phosphates to form apatite layer on surface of sample increasing final weight in this case too. Phase difference of apatite formed might be explained by presence of different ions in solution at different concentrations in accordance with leaching of ions from sample [49]. However, difference in average crystallite size of apatites formed in case of BM, BMH and BMG samples can be explained by the fact that more nucleation site decreases the average crystallite size. As HAp and BAG coatings are porous, they provide much more nucleation site then the uncoated substrate, hence lower average crystallite size, which can be seen from XRD data too. Decrease in average crystallite in case of BM and BMH samples from day 7 to 14 confirms presence of pores on surface created from corrosion. On the other hand, increase in average crystallite size for BMG samples proves better apatite formation on the surface than others. Apatite formation and corrosion is schematically represented in Fig. 10, where Ca^2+^ originated from SBF and surface of sample react with H_2_PO_4_^−^ to form insoluble apatite. During the process, OH^−^ forms and reacts with leached Mg^2+^ to form Mg(OH)_2_, which also precipitates on surface increasing resistance of the coating. Depending upon Ca^2+^ concentration in SBF solution, different phases of Ca–P form. Ca^2+^ concentration difference between supernatant of BMH and BMG after day 7 clearly gave an idea why two different apatite phases formed in BMH and BMG samples.

Cell–materials interactions require initial attachment of cells which can influence ensuing cellular and tissue responses [50]. It is reported that the adhesion and viability of cells depend on culture setting and material surface features. However, rapid degradation of Mg and its alloys leads to concomitant increase in the pH, which can be detrimental to cell adhesion and survival. This can be seen from the in vitro results of BM with least corrosion resistance. Due to absence of coating, pH rapidly increases, whereas for coated Mg alloy samples, degradation of BG is more as compared to HAp resulting less attachment, viability and proliferation of cells in the order of the BM > BMG > BMH at different time intervals of 5, 7, 14 and 21 days post incubation. Further, coated Mg alloys provide conducive atmosphere for cell connection and expansion. Any protective coatings prevent the entry of water and electrolyte [51], leading to slower corrosion of Mg alloy substrate vis-à-vis diminishing the diffusion rate of OH^−^ from Mg surface to the medium. As a result, the rise of pH of solution neighbouring the Mg sample, which in turn helps to surface attachment of numerous cells and subsequent proliferation.

Underlying principle of protective effect is that, being a reactive metal, bare Mg will act in response with water, precipitation of Mg(OH)_2_ on the surface of Mg and consequent release of H_2_ gas. It is assumed that the accrued Mg(OH)_2_ layer on Mg prevents dissolution by avoiding mass diffusion between Mg and the solution [52]. Resultant Mg(OH)_2_ is changed to more soluble MgCl_2_ by Cl^−^ [53], which releases Mg^2+^ into the solution. Mg^2+^ release depends upon severity of Mg corrosion. Accordingly, in the present study, the release of Mg^2+^ in cell culture is used to judge protective effect of HAp and BG coating on Mg substrate. It has been observed that there was low release of Mg^2+^ from HAp and BG coating compared to BM throughout the incubation period, leading to less corrosion in coated samples. On the other hand, adhesion strength also plays a vital role as corrosion resistance [54].

ALP activity and ARS assay are important tools to calculate mineralization upon implants. Cells resemble an orderly, sheet-like structure when there are considerable levels of mineralization. During the entire period of culture, prerequisite nature of samples includes rising ALP activity, intensity of Alizarin Red staining and stressed actin arrangement of cells upon samples. In the present study, HAp and BG coated samples shows favourable results in comparison to bare Mg alloy. Likewise, images of BMH show unsystematic deposition of actin-stress fibres together with dense cell colony and to some extent on BMG too. In general, in vitro analysis illustrates considerable perfection of sample properties of HAp and BG coated samples with respect to biocompatibility, cell viability and proliferation and osteoconductivity. Side by side, BMH implant is a notably better choice than BMG implant. In vivo biocompatibility can only be assessed by observing nature and magnitude of inflammation of neighbouring soft tissue reaction in presence of any foreign material. In the present study, although lesser vascularization, fibrous tissue and presence of mononuclear cells in the histological figures are observed, neither significant inflammatory reaction nor formation of gas cavities around the implantation site of bone is pronounced. This advocates that the implants are well accepted in vivo indicating a promising biodegradable implant material.

Radiographic evaluation of implants is a non-invasive technique to assess the position of implant during the healing process. In the in vivo test, radiologically a significantly higher degradation of BM was observed as compared to BMH and BMG implant. There was moderate osseous growth in defect site in BM, whereas more bone formation was observed in coated implants. Enhanced bone formation might be owing to release of Mg ions, because high Mg concentration is essential for bone cell activation [55]. Similar experiment with Mg–Ca pins in bone defect model shows better activity of osteoblasts and osteocytes around the implant [56]. Moreover, it could be expected that enhanced local pH surrounding the implanted area due to gradual corrosion of Mg alloy provides a favourable environment for mineralization. In both coated groups, implants within bone were under the process of gradual degradation as observed by reducing diameter of implant and new bony tissue ingrowths over defect area. This might be due to gradual degradation of Mg alloy from the coated implants. Moreover, during the entire healing process, no gas bubbles are observed owing to gradual release of Mg ions from coated implants during degradation [55, 56]. In the present study, radiological findings can be corroborated with the fluorochrome labelling results. Tetracycline, a bone specific marker, was used for quantifying the amount of new bone formation in the defect area. Tetracycline is deposited in any fracture site during the active mineralization process. BM implant at 2 months showed bone formation mainly at the middle and partially in peripheries, as marked by the presence of golden yellow fluorescence. In BMH and BMG implant group, the intensity of golden yellow fluorescence was more prominent at periphery with wide deep area of new bone formation. The findings of fluorochrome labelling can also be compared with histological results. No noticeable inflammatory effects are observed surrounding the implants indicating biocompatible nature of the implants. In bare Mg alloy, histological section depicted a bony matrix with abundance Haversian canal, bony lacunae and few osteoclasts along with lesser angiogenesis towards medullary region. In BMH, implant showed bony lamellae characterized by well-developed Haversian system, canaliculi and resorption of bone in pericortical areas signifying presence and delineation of osteogenic cells. In BMG, implant group depicted a large number of osteocytes, osteoclasts and osteoblast proliferation. New bone formation around the bare Mg implants at 2 months might be due to stimulating effect [55, 57]. At 2 months, HAp and BG coated samples depicted more presence of osteoid surface. The explanation for this improved osseous tissue regeneration rates adjacent to these coated implants are due to osteo-proliferative effect of calcium phosphate and BG coating of Mg implants. As the corrosion of the coated implants happens relatively slowly, the corrosion products can be safely eliminated from the body system either through absorption by the adjacent tissues or local blood circulation which corroborated the findings of reduced bone growth caused by magnesium deficiency [58].

SEM examination during the post-surgical period demonstrates better amalgamation of material with the host bone while validating infiltration of osteogenic cells and ultimately resulting into evidence of mineralized matrix. There was no visible interfacial gap between the bone and implant in BMH group. This might be due to invasion of osteoblasts towards the implant structure. For the BMG implant, interfacial gap is more pronounced which might be due to slow invasion of osteoblasts.

Histology of heart, kidney and liver was carried out to assess whether any changes happen in cellular level or not. The results established that no apparent pathological lesions were seen after 2 months of experimentation, indicating safe degradation of implants in vivo and will not create any detrimental effects.

Immune reactivity of the animals implanted with the materials (BM, BMH and BMG) post surgery and normal/control animals (without any implant) was verified by quantifying the amount of IL-2, IL-6 and TNF-α cytokine secretion. Study of host-implant inflammatory response was monitored for initial 2 months post surgery and the results showed that at first 2 weeks, amount of IL-2, IL-6 and TNF-α cytokine secretion was increased preferably because of initial host-foreign body reactions but at the end of 8 weeks, it was comparatively decreased to its baseline quite similar to control group. Comparing with the positive control group, three types of materials showed a varied reactivity, starting from highest response against BM implant followed by BMG and BMH implant. In the first 2 weeks, concentrations of IL-2, IL- 6 and TNF-α were found to be drastically higher than the control animals, likewise, from 4 weeks up to the end of the experiment (i.e. 8 weeks), secretions of cytokines (IL-2, IL-6 and TNF- α) were reasonably decreased down to its normal range. Thus, at the end of 2 months, unlike BM, neither BMG nor BMH implant materials showed any kind of marked immune reactions post surgery, in comparison to the control group.

## Conclusions

Present investigation focuses on development and detailed characterization of a new Mg–Zn–Ca alloy (BM) with and without HAp (BMH) and BG (BMG) coating deposited using air plasma spraying. Electrochemical experiments demonstrated relatively better corrosion resistance of BMH and BMG compared to BM. Among the samples, BMG exhibited lowest *I*_corr_ and *E*_corr_ suggesting its superior in vitro corrosion resistance than BMH. In addition to improved corrosion resistance, coatings clearly enhanced the apatite precipitation ability on present Mg alloy samples. Detailed in vitro cell–materials interaction experiments demonstrated that both BMH and BMG samples induces osteogenesis. In vivo trials on mature New Zealand white rabbits revealed no measurable adverse effects on heart, kidney and liver and immune response suggesting their application potential. BMG implants resulted in accelerated new bone formation, which corroborates our in vitro observations. Overall, our results show that plasma spraying of HAp and BG on this new Mg alloys can be effectively used to control rapid degradation under in vivo conditions.
